# Daily, Weekly and Monthly Variation in Lunch Time Calories

**DOI:** 10.23889/ijpds.v8i3.2288

**Published:** 2023-09-11

**Authors:** Anya Skatova, Neil Stewart, Edward Flavahan

**Affiliations:** 1 Bristol Medical School, University of Bristol; 2 Warwick Business School, University of Warwick; 3 Behavioural Insights Team; 4 Nottingham Business School, University of Nottingham

## Introduction & Background

Despite the level of attention that healthy and unhealthy eating receive from academic research, policymakers and the wider public, objective data on food consumption is limited. This is because studies of individual eating patterns using food diaries are subject to underreporting, particularly by people who are overweight. For example, the UK population is estimated to consume between 30% to 50% more calories than they report in surveys. New data sources such as office canteen ordering systems and individual records of supermarket transactions recorded through supermarket loyalty or bonus cards offer larger and potentially more robust data on real world individual eating behaviours.

## Objectives & Approach

We used 2,831,403 machine-recorded ‘meal deal’ transactions from 205,781 individuals over the course of one year from one of the UK’s largest suppliers of lunch time foods to investigate whether there is a relationship between patterns of choice and higher calorie consumption. A meal deal comprises three items; a main (e.g., a sandwich or a salad), a snack (e.g., crisps, fruit or a chocolate bar) and a drink (e.g., a smoothie or a bottle of water). In contrast to diary studies or aggregate transactional data from supermarkets, our dataset included “meal deal’ purchase which is highly likely to be made by an individual for their own consumption or soon afterwards.

## Relevance to Digital Footprints

Lunch time food consumption can reflect the overall diet the individual is exposed to, helping to understand population level patterns of people’s food choices through a type of digital footprints data - shopping history records.

## Results

Controlling for gender, general index of variety in the choice of lunch food items, income and education, we found that individuals who vary in their calorie consumption most across the time of day, day of the week, and month of the year are the individuals who consume the greatest number of calories overall. These time sensitivity effects are large, together explaining a substantial amount of variance in calorie consumption. Time sensitivity effects are strongly correlated across all three time scales suggesting they measure a stable underlying trait.

## Conclusions & Implications

Individuals vary calorific composition of their lunch over time of the day, day of the week and month of the year by 100 calories per meal between highest and lowest in sensitivity which is about 9% of the recommended amount of lunchtime calories. Those whose consumption varies the most with time consume the most calories, independently of income and gender. The variation in calories at all three time scales demonstrates the properties of an individual disposition. These findings can be used to understand why and when people make unhealthy food choices.

